# 

**Published:** 1896-01-25

**Authors:** 


**Notices of Births, Deaths, and Marriages** are inserted in this column at a charge of 2s. 6d. for an announcement not exceeding four lines.

**Notice to Advertisers and Subscribers.**

All Advertisements, Orders for Copies of The Hospital and Business Communications should be addressed to **THE MANAGER, (Not to The Editor),** The Hospital, 428, Strand, London, W.C.

The Cost of Subscription, post free, is as follows:—Per week, 2½d.; per quarter, 2s. 8d.; per half-year, 5s. 3d.; per annum, 10s. 6d. Foreign:—Per Annum, 15s. 2d.; payable, in all cases, in advance.

### NOTICE TO CORRESPONDENTS.

All MS., Letters, Books for Review, and other matters intended for the Editor should be addressed The Editor, The Lodge, Porchester Square, London, W.

The Editor cannot undertake to return rejected MS., even when accompanied by stamped directed envelope.

**"Burdett's Hospital Annual," 1895.**

Sixth year of publication. 950 pp. Scarlet cloth, gilt lettered. Price 5s. A most complete guide to British, Colonial, Indian, and American Hospitals, Dispensaries, Nursing and Convalescent Institutions, and Asylums. A few complete sets of the preceding five issues of the "Annual" may still be obtained on application to the Publishers, The Scientific Press (Limited), 428. Strand, London, W.C.

**NOTICE.—Vol. XVIII. of "The Hospital" (April to September, 1895), in handsome cloth binding, is on sale at the Office of this Journal. Price 7s. 6d. Cases for binding the Numbers contained in this Volume, 1s. 6d. Index to Vol. XVIII., 2½d., post free. The Monthly Part for February will be ready next week, price 6d., by post, 8d.**

### SCRAPS AND GLEANINGS.

OVER £4,000 have now been subscribed to the "Asile International Russe" at St. Petersburg.

A DONATION of £50 has been sent to the Meath Hospital and County Dublin Infirmary from Wellington Darley, Esq.

THE Royal Sea Bathing Infirmary, Margate, has received £1,000 from the executors of Miss M. A. Robinson.

THE Saddlers' Company have given no less than £5,500 during last year in subscriptions and donations to charitable institutions.

WE are glad to notice an increase in the Hospital Sunday collection at Aberdeen, this year's collection being £143 in excess of last year.

THE French Army Ambulance Corps are to be provided with incandescent lamps and pocket batteries for use on the battlefield at night.

THE Royal Hospital for Incurables at Putney has benefitted through the will of the late Mrs. Mary Ann Jewell Allcroft to the extent of £1,000.

THE bazaar held at Manchester for the funds of the National Society for the Prevention of Cruelty to Children brought in about £10,000 in support of the scheme.

A SUM of £3,000 has been received from an anonymous donor in aid of the building fund of the Grosvenor Hospital for Women and Children, Vincent Square, Westminster.

H.R.H. THE PRINCE OF WALES will visit Brighton on February 15th in connection with the movement for the enlargement of the Sussex County Hospital, to which scheme we have already referred.

DURING Christmas the Empress Frederick paid a round of visits to the hospitals at Berlin. No fewer than 156 children at the Emperor and Empress Frederick's Hospital received gifts from her Imperial Majesty's hands.

THE Queen has sent a present of game for the patients and nursing staff of the Middlesex Hospital. Her Majesty has sent similar gifts to St. Mary's Hospital, Paddington, and the Royal Westminster Ophthalmic Hospital.

A DONATION of £25 has been sent to the North-Eastern Hospital for Children by the Dowager Lady Penrhyn, who has responded to Lord Frederick Fitzroy's appeal for nine such subscriptions to the funds of this charity.

THE important scheme for building the Bedford County Hospital is again being brought before our attention, as a sum of £8,000 has yet to be raised to complete the requisite £22,000 required for the execution of the scheme.

THE treasurer of Guy's Hospital has received £221 from Mr. Cosmo Bonsor, M.P., as the proceeds of a concert, the expenses of which were borne by the staff of the South-Eastern Railway Company, of which Mr. Bonsor is chairman.

A LEGACY of £500 has been received at the Glasgow Cancer Hospital, from the trustees of the late Charles Kidston, and £100 from an anonymous donor. Over £3,000 is still required to finish the extended buildings of the new hospital.

THE first annual collection of the Leeds Medical Charities held in the schools of the city has been highly successful. The working classes, by means of similar collections weekly, have, since 1887, given £30,000 to the local medical charities.

FIFTY pounds and forty pounds respectively have been received by the secretary of the Home for the Dying at Upper Avenue Road, N.W., by two generous donors who have recognized the urgency of increased financial help for this home.

THE annual meeting of the subscribers of the Aberdeen Ophthalmic Institution was held recently. Since the removal to more extensive premises over 560 new cases have been treated, compared with the same period previous to the move.

ANNUAL subscriptions of 5s. and upwards are urgently solicited by the council of the Great Northern Central Hospital, Holloway, the income of the hospital from this source being quite inadequate to the demands made upon it. By asking so small an annual subscription at our hands it makes it possible for many thousands among us to subscribe, and thus help forward the work of this admirable institution.

### THE STUDENT OF MEDICINE.

### APPOINTMENTS.

A. Back, M.A.Camb., L.R.C.P.Edin., M.R.C.S.Eng., appointed Medical Officer and Public Vaccinator for the Fifth District of the Aylsham Union. G. C. Barker, M.A.Camb., M.D.Brux., L.R.C.P.Lond., M.R.C.S.Eng., appointed Honorary Physician to the Brighton and Hove Dispensary. W. Berry, J.P., F.R.C.S.I., D.P.H., appointed Medical Officer of Health and Superintendent of the Sanatorium, County Borough of Wigan. A. R. Darley, M.D., B.Ch., appointed Medical Officer of Health for the Daventry Rural District. G. W. Davis, M.D.Durh., L.R.C.P.Lond., M.R.C.S.Eng., appointed Certifying Factory Surgeon for Sidcup. Miss R. C. Despard, M.B.Lond., appointed a Junior Assistant Medical Officer at the Holloway Sanatorium Hospital for the Insane, Virginia Water. Dr. Donelan, appointed Physician to the Auxiliary Workhouse at Portrane. W. L. Fry, M.A., B.M., B.Ch.Oxon., appointed House Physician to the Radcliffe Infirmary, Oxford. F. W. Garrad, M.B., B.C.Cantab., appointed House Surgeon to the Warneford and South Warwickshire Hospital, Leamington. T. W. H. Garstang, B.A.Oxon., M.R.C.S.Eng., appointed Medical Officer of Health for the Biddulph Urban District. G. G. W. Henry, M.R.C.S.Eng., L.R.C.P.Lond., appointed Medical Officer of

THE STUDENT OF MEDICINE—*continued.*

Health for the Minehead Urban District. W. A. Higgs, M.R.C.S., L.R.C.P., appointed Medical Officer for the Castle Combe District of the Chippenham Union. A. Hodge, M.R.C.S. Eng., L.R.C.P.Lond., appointed Resident Medical Officer to the Chorlton-on-Medlock Dispensary, Manchester. J. S. Holden, M.D., appointed Medical Officer of Health to the Belchamp and Melford Rural District Council. H. T. Hollings, L.R.C.P., L.R.C.S.Edin., appointed Medical Officer for the Holme-on-Spalding Moor District. R. P. Jack, M.B., C.M.Edin., appointed Medical Officer of Health to the Dalziel Parish Council. W. E. Johnstone, M.A., M.B., C.M.Edin., appointed House Surgeon to the Darlington Hospital and Dispensary. J. H. Lamb, M.B., C.M.Edin., appointed House Physician to the Royal Berks Hospital, Reading. J. Ferguson Lees, M.B., C.M.Glasg., appointed House Surgeon to the Hartlepool Hospital. E. P. Manby, M.D., D.P.H.Camb., appointed Assistant Medical Officer of Health for Liverpool. W. R. Martine, M.B., C.M.Edin., appointed Medical Officer and Vaccinator to the Athelstaneford Parish Council, Haddington, and Medical Officer and Vaccinator to the Morham Parish Council, Haddington. A. Miles, M.D., F.R.C.S Lond., appointed Surgeon to Leith Hospital. J.N. Phillips, L.R.C.P.Lond., M.R.C.S.Eng., appointed Medical Officer of Health for the Cannock Rural District. W. T. G. Pugh, M.B., B.S.Lond., M.R.C.S., L.R.C.P., appointed House Physician to the North-Eastern Hospital for Children, Hackney Road. B. Saunders, M.B., C.M.Aberd., appointed Junior House Physician to the North-Eastern Hospital for Children, Hackney Road. Dr. Shearer, appointed Medical Officer for the Gotham District of the Basford Union. A. Strange, appointed Assistant Medical Officer to the Whitechapel Union Infirmary. Dr. Swan, appointed Medical Officer for the Drum District of the Cootehill Union. Dr. Whitwell, appointed Medical Officer for the No. 3 District of the Market Harborough Union. C. Wills, M.R.C.S.Eng., appointed Medical Officer of Health to the Blyth and Cuckney, Clown and Kiveton Park Rural District Councils. R. S. Olver, M.R.C.S., &c., appointed House Surgeon to the Derbyshire Royal Infirmary. W. E. L. Horner, M.B.Lond., appointed House Physician to the Derbyshire Royal Infirmary.

### VACANCIES.

The following vacancies are announced this week, the amount of salary in each case being given after the name of the institution:—

**Resident Medical Officers.**

Bradford Infirmary.—Dispensary Surgeon; unmarried; doubly qualified. Salary £100 per annum, with board and residence. Applications, endorsed "Dispensary Surgeon," to William Maw, Secretary, by January 27th.

Great Northern Central Hospital, Holloway, N.—Junior House Surgeon. Board, lodging, and laundry provided. Applications to the Secretary by January 27th.

North Staffordshire Infirmary and Eye Hospital, Hartshill, Stoke upon-Trent.—Assistant House Surgeon. Board, washing, and apartments provided. Applications to the Secretary by January 27th.

Scottish Prison Service.—Resident Medical Officer. Salary £250, with a house. Applications to the Secretary of the Prison Commission for Scotland, 6, Rutland Square, Edinburgh, by January 31st.

Tower Hamlets Dispensary.—Resident Medical Officer. Salary £120 per annum, with furnished rooms, coals, gas, and attendance. Applications to the Secretary, Mr. D. F. Matheson, Tower Hamlets Dispensary, White Horse Street, Stepney, by February 1st.

**Non-Resident Medical Officers.**

Grosvenor Hospital for Women and Children, Vincent Square, Westminster, S.W.—Assistant Physician; must be Member of the Royal College of Physicians. Applications to the Secretary by January 29th.

Middlesex Hospital Medical School.—Lecturer on Anatomy and a Lecturer on Physiology. Applications to the Dean of the Medical School, Cleveland Street, W., by February 17th.

National Hospital for the Paralysed and Epileptic (Albany Memorial), Queen's Square, Bloomsbury.—Assistant Physician; must be a Graduate in Medicine of a University and Member or Fellow of the Royal College of Physicians of London. Applications to B. Burford Rawlings, Secretary-Director, by January 31st.

Royal Free Hospital, Gray's Inn Road.—Assistant Physician to out-patients; must be F. or M.R.C.P.Lond. Applications to the Secretary by January 25th.

University of London.—Registrarship. Salary commences at £800, and rising by annual increments to £1,000 per annum Applications to Arthur Milman, M.A., LL.D., Registrar, University of London, Burlington Gardens, W., by January 25th.

West London Hospital, Hammersmith Road, W.—Assistant Physician; must be Fellow or Member of the Royal College of Physicians of London. Applications to R. J. Gilbert, Secretary-Superintendent, by February 5th.

GREAT NORTHERN CENTRAL HOSPITAL,
Holloway, N.

### ROYAL NATIONAL PENSION FUND FOR NURSES.

*President*—**H.R.H. THE PRINCESS OF WALES.**

*Chairman*—WALTER H. BURNS, Esq. *Deputy-Chairman*—HENRY C. BURDETT, Esq.

**PENSIONS. SICKNESS. ACCIDENT.**

### Total Invested Funds, £250,000.

Nurses are invited to join the Fund on account of the substantial and exceptional advantages which it offers them and which *they cannot obtain elsewhere.* The following are the chief points :—

1. **The Fund is Mutual and essentially Co-operative.**
2. **Easy Payment of Premiums.**
   Nurses can pay their premiums monthly or otherwise as best suits their convenience.
3. **The Fund is open to every Nurse.**
   Nurses can assure for Pensions of any amount commencing at any age.
4. **An Investment and Savings Bank.**
   Those entering under the returnable scale can have their premiums returned to them with compound interest, less a small deduction for working expenses. Interest allowed on deposits.
5. **Additions to Pensions.**
   Besides the Pension entered for, substantial additions thereto may be anticipated in the shape of bonuses.
6. **Sickness and Accident Assurance.**
   Policies are issued in connection with Pension policies assuring 10s., 15s., or 20s. a week in cases of incapacity from work through sickness or accident.

**Full particulars to be obtained from THE SECRETARY,**

ROYAL NATIONAL PENSION FUND FOR NURSES,

28, FINSBURY PAVEMENT, LONDON, E.C.

